# 1193. Impact of Removing Extended-spectrum Beta Lactamase Status Labeling from Culture Reports on Carbapenem Use for Non-bacteremic Patients With Urinary Tract Infections: A Multi-hospital Assessment

**DOI:** 10.1093/ofid/ofad500.1033

**Published:** 2023-11-27

**Authors:** Lourdes R Menendez Alvarado, Alice Margulis Landayan, Kelsey N Williams, Corey M Frederick, Jorge Murillo, Timothy P Gauthier

**Affiliations:** Baptist Health South Florida, Miami, Florida; Baptist Health South Florida, Miami, Florida; Baptist Health South Florida, Miami, Florida; Baptist Health South Florida, Miami, Florida; Baptist Health South Florida, Miami, Florida; Baptist Health South Florida, Miami, Florida

## Abstract

**Background:**

Reporting extended-spectrum beta lactamase (ESBL) status on microbiology results for Enterobacterales is not required by laboratory, infection prevention, or antimicrobial stewardship guidelines. Ceftriaxone (CRO) resistance may be utilized as a proxy for ESBL production. Current practice guidelines recognize select non-carbapenems as acceptable treatment options for ESBL-producing uncomplicated urinary tract infections (UTI). This project aimed to evaluate carbapenem prescribing for CRO-resistant UTIs caused by Enterobacterales before and after removal of ESBL status from microbiology laboratory results at seven sites across a large health system.

**Methods:**

This was a retrospective cohort study of adult patients treated for at least 48 hours for an ESBL-producing/CRO-resistant Enterobacterales UTI. Patients were excluded if they had a concomitant infection outside the urinary tract, a polymicrobial infection, or if treatment was started outside the hospital. The primary endpoint was the rate of carbapenem prescribing for initial definitive treatment of UTIs. Secondary endpoints included total days of therapy for initial definitive therapy with carbapenems, clinical cure rates, time to transition to oral antibiotic therapy for initial definitive therapy, rate of guideline-compliant therapy, 30-day readmission rate, rate of relapsed infection within 30 days, and 30-day all cause in-hospital mortality. This study was approved by the Institutional Review Board.

**Results:**

Of 3055 patients screened, 199 were included in the pre-group and 153 were included in the post-group. Demographics are displayed in Table 1. The rate of carbapenem prescribing for initial definitive treatment was 156 patients (78%) in the pre group, compared to 93 patients (61%) in the post group (p=< 0.01). The rate of non-carbapenem prescribing was 43 patients (22%) in the pre-group, compared to 60 patients (39%) in the post-group (p=< 0.01). There was a 40% reduction in total days of therapy for initial definitive therapy with carbapenems (620 vs. 372, p=< 0.01). Other secondary outcomes are listed in Table 2.

**Figure:**
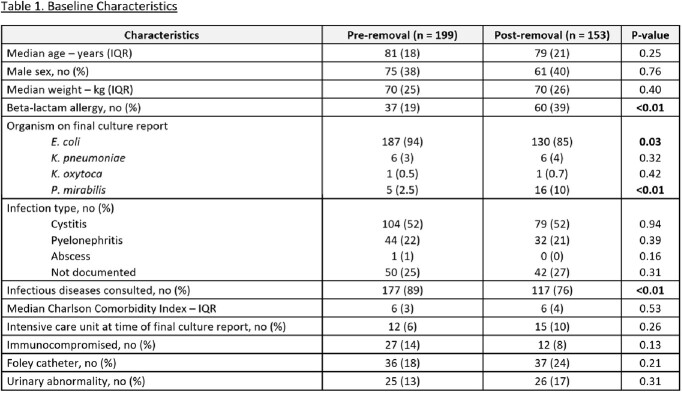

**Figure:**
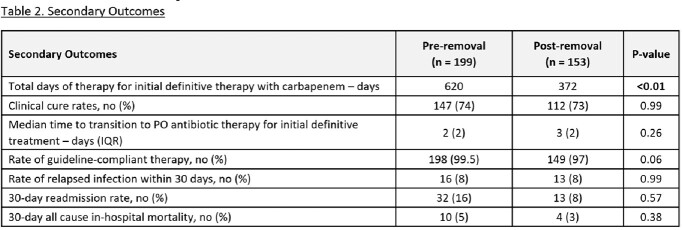

**Conclusion:**

Removal of ESBL status labels from laboratory reports may substantially reduce carbapenem use for initial definitive treatment of UTIs without impacting clinical outcomes.

**Disclosures:**

**Jorge Murillo, MD, FIDSA, FACP**, Insmed: Advisor/Consultant|Melinta Therapeutics: Advisor/Consultant|Merck: Advisor/Consultant **Timothy P. Gauthier, PharmD, BCPS, BCIDP**, Ferring: Advisor/Consultant|Firstline: Advisor/Consultant|Firstline: Writing|GSK: Advisor/Consultant|LearnAntibiotics.com & IDstewardship.com: Owner|Melinta: Advisor/Consultant|Pattern Biosciences: Advisor/Consultant|Pfizer: Advisor/Consultant

